# Second Primary Lung Cancer Associated With Family History of Lung Cancer

**DOI:** 10.1002/cam4.71431

**Published:** 2025-11-29

**Authors:** Frantisek Zitricky, Kristina Sundquist, Jan Sundquist, Asta Försti, Akseli Hemminki, Kari Hemminki

**Affiliations:** ^1^ Biomedical Center, Faculty of Medicine in Pilsen Charles University Pilsen Czech Republic; ^2^ Center for Primary Health Care Research Lund University Malmö Sweden; ^3^ University Clinic Primary Care Skåne University Hospital Region Skåne Sweden; ^4^ Department of Family and Community Medicine, McGovern Medical School The University of Texas Health Science Center Houston Texas USA; ^5^ Hopp Children's Cancer Center (KiTZ) Heidelberg Germany; ^6^ Division of Pediatric Neurooncology German Cancer Research Center (DKFZ), German Cancer Consortium (DKTK) Heidelberg Germany; ^7^ Cancer Gene Therapy Group, Translational Immunology Research Program University of Helsinki Helsinki Finland; ^8^ Comprehensive Cancer Center Helsinki University Hospital Helsinki Finland; ^9^ Division of Cancer Epidemiology German Cancer Research Center (DKFZ) Heidelberg Germany

**Keywords:** adenocarcinoma, incidence trend, proband, sibling risk

## Abstract

**Background:**

Familial clustering of initial primary lung cancer (IPLC) may be related to shared smoking habits, other environmental exposures and hereditary factors, but whether familial risk also influences the risk of second primary LC (SPLC) is not well known. We aimed to carry out a family study between first‐degree relatives on SPLCs in Sweden.

**Methods:**

Population data on Swedish family relationships and the diagnosed cancers were obtained from the national registers from 1961 to 2021. IPLC was diagnosed in 54,429 patients of whom 534 were diagnosed with SPLC. Familial risk was assessed through the standardized incidence ratio (SIR with 95% confidence interval) adjusted for several potential confounders, including sex, age, calendar period, educational level and geographic region. Familial risks were analyzed by type of proband, histology and sex. In addition, we estimated the effect of family history on the cumulative proportion of patients developing SPLC by sex and histology.

**Results:**

The estimated SIR for SPLC was 3.98 in patients without family history and 5.24 among those with a history of lung cancer in first‐degree relatives. The SIR values depended on the histology of IPLC and of SPLC, with the highest SIRs for concordant histologies. For the adenocarcinoma‐adenocarcinoma sequence, SIR estimates were 5.60 and 7.51 for non‐familial and familial patients, respectively. The familial risks were further modulated by sex and type of affected relative, with the highest SIR for females with affected mothers (9.14).

**Conclusions:**

The results showed a positive association of family history of LC with risk of SPLC on top of high risk for SPLC in non‐familial patients. The risks differed by sex, histology and type of affected relative. The data on family history of LC should alert about surveillance for SPLC and may be used in future risk stratification when eligibility for population screening is considered.

## 1 Introduction

In industrialized countries the lung cancer (LC) epidemic started after World War II, following some 20–30 years after the huge increase in tobacco smoking habits [1, 2]. The oldest cancer registry data from the USA from the year 1935 showed that male LC incidence increased about 8‐fold by 1980; the increase in female incidence was even higher but it started at a lower level and later; in 1935‐39 male incidence was 10.2 compared to 3.4 in women; 30 years later these were 64.1 and 11.1, respectively [3]. The oldest European data are from Denmark from 1943 documenting an increase in LC incidence of 5‐fold in the subsequent 30 years [4]. In US men LC incidence started to decline before 1990 and in women almost 20 years later [5]. In Europe, male LC incidence peaked earliest in Sweden in 1982, while for Swedish women the incidence culminated decades later in 2018, after the epochal crossing of the male and female LC incidence rates [6].

In Swedish men, squamous cell carcinoma (SCC) was the most common histological type of LC, but it was overtaken by adenocarcinoma in the 1990s and then SCC declined to 20% of all LC. In Swedish women, adenocarcinoma has been the main type throughout [7]. The share of small cell LC has been declining to 10% of all, equal to undifferentiated (large cell) LC. The dominant role of adenocarcinoma histology in LC has been a global trend; by 2020 it was the most common female histology in all and male histology in most countries [8]. One likely explanation for this trend is the common decline in the prevalence of smoking, as adenocarcinoma is less associated with smoking than the other LC histologies [8]. A consequence of declining smoking will be that the etiological role of smoking as the cause of LC will decrease and assumably the role of other environmental and genetic factors, including familial risk, will become more prominent, as they already appear to be in populations with low smoking prevalence [9, 10, 11]. Another consequence of declining smoking and the relative increase in less fatal adenocarcinoma compared to the aggressive histological types will be an improvement in survival in LC, for which earlier diagnosis and novel treatments are important contributors [12, 13, 14, 15].

The etiology of independent second primary LC (SPLC) is related to the underlying disease and its treatment and may be shared by first LC, including smoking, other environmental, familial and hereditary factors, and additionally field cancerization is possibly a unique mechanism for SPLC [16, 17]. Familial risk of LC between first‐degree relatives is about 2.0, which is at the level of many common cancers [18, 19]. However, in populations with a history of smoking, familial risk is likely to be partially due to shared smoking habits between family members, as it has been shown that unrelated spouses share risk of LC to a much greater extent than sharing between any other cancer [20]. Similarly, a recent Swedish study showed high concordance for LC risk for maternal compared to paternal half‐siblings, explained by cohabitation of only maternal half‐siblings [21].

In the present study, we quantify familial risk for SPLC according to the histology of the first LC. The familial risk for SPLC has been the subject of only a few earlier studies [19, 22]. Understanding the association between family history and SPLC is helpful guidance for follow‐up of patients with IPLC and in the future as a component in risk‐stratification models to preselect high‐risk individuals eligible for LC screening.

## 2 Patients and Methods

### 2.1 Lung Cancer in Sweden 2009–2023

Estimated smoking prevalence in Sweden since 1950 and sex‐and histology‐specific LC incidence rates since 1960 were reported in Supporting Information in a recent LC paper [10]. While adenocarcinoma and SCC were diagnosed from 1960 onwards, small cell (PAD 186) and large cell (PAD 196) carcinomas were first diagnosed in the mid 1980s as separate entities from earlier unspecified/large cell cancer. The shorter follow‐up times for small cell and large cell carcinomas reduce somewhat case numbers for these rare histologies.

Sweden has established a national system of quality registers on many diseases and conditions, including cancer. The LC quality register has operated since 2002 with interactive data available from 2009 to 2023 https://statistik.incanet.se/Lunga/. Among male non‐small cell LC (NSCLC) patients between 2009 and 2023 the proportion of never smokers increased from 7% to 11%, and that for quitters from 51% to 55%; the proportion of smokers declined from 42% to 33%. Among female NSCLC patients the proportion of never smokers remained at about 17%, the proportions of quitters and smokers were close to each other at about 40% but diverged until 2023 to 50% for quitters and 33% for smokers.

NSCLC in men increased from 80% to 82% (adenocarcinoma 42%–53%) of all LC, compared to women from 78% to 79% (adenocarcinoma 47%–60%). For male NSCLC stage IV accounted initially for 54% of all and it declined to 45% towards the end; stage IA increased from 10% to 23%. For female stage IV LC, the percentage at each time point was a few % units (varying between 3% and 5% units) below the male ones, ending at 42%; conversely the curve for stage IA ran (5%–8% units) on top of the male curve ending at 28%.

For NSCLC patients at stage IA‐IIB and WHO performance status (0–2) planned curative surgery or radiation increased from 50% to over 90%. For NSCLC patients at stage IV (excluding SCC) the plan for active treatment after epidermal growth factor receptor (EGFR) test increased from null to 90%. For NSCLC patients at stage IV, with WHO performance status 0–2 (i.e., 0 normal activity without symptoms; 1, capable of self‐care but unable to work; 2, ambulatory and capable of all self‐care but unable to carry out any work activities) palliative medical treatment for the wide‐spread disease increased from 83% to 95%.

## 3 Methods

Parent‐offspring relationships were available in the multigeneration register. It includes essentially complete families that are registered after each childbirth [23]. Parents were individuals born before 1932 and offspring were their children [23]. Siblings could be identified through common parents in the population born after 1931, the oldest of whom reached age 89 years by 2021. All family and cancer linkages were done through the unique personal identification number replaced by a serial number to secure people's integrity. The study was restricted to persons born in Sweden with both parents identified. The follow‐up started on the date of birth or the beginning of the study (1st January 1961), whichever came later. The follow‐up was terminated at the time of death, emigration, LC diagnosis or the end of the study (29th December 2021), whichever came earliest.

LC was defined through ICD‐7 code 1621‐“LC specified as primary.” For any reported cancer, the cancer registry makes an effort to include only primary cancers, and we thus presume that multiple LCs are independent primaries [24]. Nevertheless, we included only SPLCs diagnosed at least 1 month after initial primary lung cancer (IPLC). In the Swedish cancer registry 98% of cancers are histologically verified. The main histological types were available throughout the whole study period. The analysis was restricted to the four main histologies of IPLC, including adenocarcinoma (pathologic‐anatomic diagnosis, PAD 96), squamous cell carcinoma (PAD 146), small cell carcinoma (PAD 186, available since 1986) and large cell carcinoma (PAD 196). PAD 196 coded for “unspecified” type before 1986, including both small cell and large cell carcinoma; we include all 196 labeled tumors in the large cell carcinoma category.

We first calculate the relative risk of SPLC compared to that for IPLC. This was done using the standardized incidence ratio (SIR) as the ratio of the observed number of SPLC cases compared to the expected number calculated for IPLC, standardized for sex, age (5‐year groups), calendar period (10‐year groups), educational level (< 10, 10–11 years, 12 years, college < 3 years, university graduate) and geographic region (north, south and three largest cities). The 95% confidence intervals (CIs) were calculated assuming that observed rates follow a Poisson distribution, the main assumption for the SIR application.

Familial risks of SPLC were calculated for a person (case) when his parent or sibling (proband) was diagnosed with LC. Risks for SPLC were estimated based on SIR as the ratio of the observed number of familial cases in offspring at risk (with LC diagnosed in parents or siblings) compared to the expected number of cases [10], which was calculated based on IPLC incidence in the offspring population without a first‐degree relative (parent or sibling) with LC. SIRs were standardized as above.

The rate ratio (RR) was calculated between incidence rates of SPLCs of familal and non‐familial group. RRs were calculated for all (standardized for sex) and specific sexes and they were standardized for age group (< 60, 61–74, 75 and older). The confidence intervals for RR were estimated by bootstrapping (as a relatively small dataset was available), for which the variability of both observed and expected rates was considered. Specifically, the subgroups of IPLC patients and the reference population were 1000 times resampled with replacement, and RR was calculated for each iteration, obtaining a bootstrap distribution for the RR metric with 2.5th and 97.5th percentiles, corresponding to the lower and upper bounds of the 95% bootstrap confidence interval.

We use cumulative proportions (CumP) of SPLC to represent the combined proportions (or probabilities) of SPLC diagnosed by a defined follow‐up time, accounting for competing risks. CumP with CIs were estimated as a cumulative incidence function using the “cmprsk” package in R. Death and diagnosis of lung tumor not meeting criteria for primary LC were treated as competing risks.

We considered SIRs to be significantly different when their 95% CIs were nonoverlapping.

All statistical analyses and data visualization were done using SAS and R (version 4.4.0).

## 4 Results

We followed 54,429 patients in the offspring generation diagnosed with IPLC of the four main histologies, starting 1 month after the initial diagnosis. A total of 534 patients were diagnosed with SPLC, 321 (60%) after the first adenocarcinoma, 120 (23%) after SCC, 28 after small cell carcinoma and 65 after large cell carcinoma (Table 1). The most common SPLC was adenocarcinoma, 61% of all, and the percentage was 77 for SPLC of adenocarcinoma histology after the first adenocarcinoma (as IPLC).

**TABLE 1:** Case numbers of second primary lung cancer (LC) in offspring generation by histology.

First primary LC type	Second primary LC type				
	All	Adenoncarcinoma	Squamous cell	Small cell carcinoma	Large cell carcinoma
All LC	534	326 (61.0%)	100 (18.7%)	48 (8.9%)	60 (11.2%)
Adenocarcinoma	321	247 (77.0%)	32 (10.0%)	22 (6.9%)	20 (6.2%)
Squamous cell carcinoma	120	36 (30.0%)	45 (37.5%)	17 (14.2%)	22 (18.3%)
Small cell carcinoma	28	11 (39.3%)	13 (46.6%)	0 (0%)	5 (17.9%)
Large cell carcinoma	65	34 (52.3%)	10 (15.4%)	9 (13.8%)	13 (20.0%)

Relative risk (SIR) of SPLC was about 4‐fold higher than that for IPLC cancer (Table 2). However, when a first‐degree relative was diagnosed with LC the risk increased to 5.24 for all LC and to 5.95 for adenocarcinoma. After first adenocarcinoma, familial risk for second adenocarcinoma was 7.51. We estimated also relative risk for SPLC as sex‐ and age‐ standardized RR of familial and non‐familial SPLC. Overall RR was 1.34, rising to 1.44 if IPLC was adenocarcinoma. For concordant adenocarcinoma the RR was 1.43. SIR for familial concordant SCC of 4.18 was not increased and results are not shown.

**TABLE 2:** Numbers and risk of second primary lung cancer (LC) based on histology and familial history.

Sporadic: no FDR (parent/sibling) with LC									
First LC type	All LC			Adenocarcinoma			SCC		
	*N*	SIR	95% CI	*N*	SIR	95% CI	*N*	SIR	95% CI
All LC	425	3.98	[3.61–4.38]	260	4.42	[3.90–4.99]	81	3.83	[3.04–4.76]
Adenocarcinoma	254	3.95	[3.48–4.46]	196	5.60	[4.84–6.44]	23	1.87	[1.18–2.80]
SCC	97	4.30	[3.49–5.25]	29	2.45	[1.64–3.51]	39	8.05	[5.72–11.00]
Small cell	21	2.87	[1.78–4.39]	8	2.02	[0.87–3.98]	10	7.15	[3.43–13.14]

Familial: ≥ 1 FDR with LC (parent/sibling)										
First LC type	All LC					Adenocarcinoma				
	*N*	SIR	95% CI	RR	95% CI	*N*	SIR	95% CI	RR	95% CI
All LC	108	5.24	[4.30–6.33]	1.34	[1.08–1.65]	67	5.95	[4.61–7.56]	1.35	[1.01–1.73]
Adenocarcinoma	68	5.48	[4.25–6.94]	1.44	[1.10–1.85]	52	7.51	[5.61–9.85]	1.43	[1.04–1.94]
SCC	20	4.18	[2.55–6.46]	1.02	[0.58–1.56]	6	2.38	[0.87–5.17]	1.00	[0.18–2.22]
Small cell	8	5.75	[2.48–11.33]	1.96	[0.66–4.34]	3	4.01	[0.83–11.70]	1.91	[0–6.66]

*Note:* Follow‐up started 1 month after diagnosis. Reference: Rates of primary LC in patients without personal or familial history of LC.

Abbreviations: FDR, first degree relative; SCC, squamous cell carcinoma.

The risk of SPLC was also evaluated by sex and type of proband (Table 3). In the non‐familial group, female risk (SIR = 4.45) was higher than male risk (SIR = 3.35). The SIR for males with an affected father was 5.34 (RR 1.62) and with an affected mother it was 5.11 (RR 1.46). For females with a positive maternal history the SIR was 9.14 (with a significant RR of 2.05).

**TABLE 3:** SIR for SPLC in offspring generation by family history and sex.

Proband	Males					Females				
	*N*	SIR	95% CI	RR	95% CI	*N*	SIR	95% CI	RR	95% CI
No FDR	159	3.35	[2.85–3.92]	1	Ref.	266	4.45	[3.93–5.02]	1	Ref.
Father	13	5.34	[2.84–9.13]	1.62	[0.81–2.62]	13	4.03	[2.15–6.89]	0.93	[0.43–1.51]
Mother	5	5.11	[1.66–11.93]	1.46	[0.32–3.06]	18	9.14	[5.42–14.45]	2.05	[1.18–3.13]
Sibling	15	3.73	[2.09–6.15]	1.15	[0.63–1.82]	34	6.05	[4.19–8.46]	1.43	[0.95–1.95]

**FIGURE 1:**
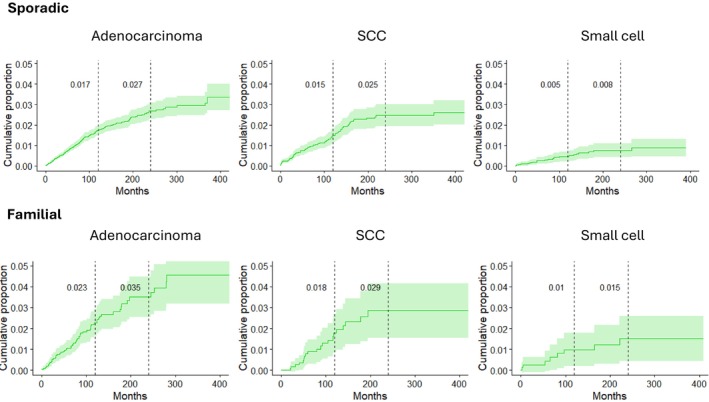
Cumulative proportion (CumP) of SPLC by IPLC histology and family history (1961–2021). Family history was positive when at least one first‐degree relative was diagnosed with IPLC.

CumP of SPLC was plotted according to the follow‐up time starting one month after diagnosis of IPLC and histological type in non‐familial (top) and familial cases up to more than 30 years of follow‐up (Figure 1). For non‐familial adenocarcinoma, SPLC CumP reached 0.27 in 20 years with some increase even to the end of the follow‐up. For SCC the 20‐year CumP was almost identical, 0.025, but hardly any increase took place at longer follow‐up. For small cell LC the 20‐year CumP was only 0.008. For familial LC 20‐year CumPs were higher: 0.035 for adenocarcinoma, 0.029 for SCC and 0.015 for small cell LC.

**FIGURE 2:**
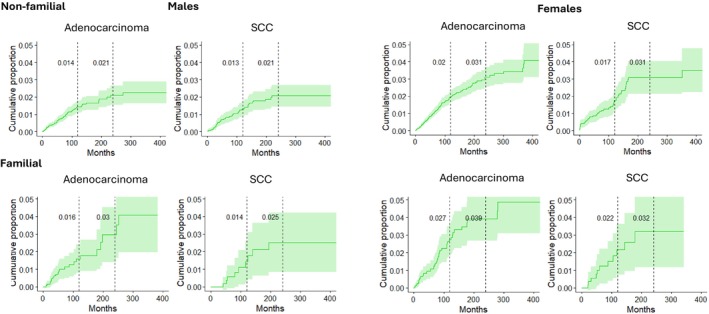
Cumulative proportion (CumP) of SPLC by IPLC histology, sex and family history (1961–2021). Family history was positive when at least one first‐degree relative was diagnosed with IPLC.

CumP of SPLC was plotted according to the follow‐up time in non‐familial (top) and familial cases, men left, women right (Figure 2). For non‐familial male adenocarcinoma, SPLC CumP reached 0.21 in 20 years of follow‐up; for familial cases CumP was 0.030. For non‐familial SCC the CumP was identical to adenocarcinoma patients and reached 0.025 in familial patients. Female non‐familial adenocarcinoma patients' CumP at 20 years was 0.031, increasing to 0.039 in familial cases. For SCC CumPs were only slightly lower (0.032) for familial cases.

## 5 Discussion

Identification of risk factors for SPLC is needed because screening for early diagnosis of SPLC is possible as shown in computed tomography (CT)‐based screening studies and some randomized trials [16, 25, 26, 27]. The results have stimulated plans for population‐based screening of risk groups in many countries [26, 28, 29]. Thus evidence‐based criteria are needed for a just prioritization of individuals for the resource‐intensive screening programs (see e.g., ERS/ESR factsheet). The present results show first that the relative risk (SIR) for SPLC compared to IPLC is substantial, about 4.0 in non‐familial cases, and it increased to 5.60 for SPLC of adenocarcinoma histology after first adenocarcinoma; SIR for SPLC of SCC histology after first SCC was even higher, 8.05. Family history was important in adenocarcinoma increasing the SIR to 5.48 (RR between familial/non‐familial was 1.44) and after first adenocarcinoma reaching an SIR of 7.51 (RR 1.43). Familial risk was highest 9.14 (RR 2.05) for daughters of affected mothers which is probably explained by the common adenocarcinoma history in Swedish women diagnosed with LC.

The observed additional risk (RR) of family history on SPLC over the risk of non‐familial SPLC was 1.34 for all SPLC and it was 1.44 for second adenocarcinoma. These are clearly smaller than the RRs of about 2.0 for IPLC [10]. In that study by us on familial risk for IPLC the highest RRs were noted for concordant adenocarcinoma and SCC, both with SIRs of 2.0 while for discordant familial cancer SIRs were around 1.5 [10]. The smaller influence of family history on SPLC incidence is expected because the familial RR for SPLC is measured on top of the high background risk in non‐familial cases of 4.0 for all and of 5.6 for adenocarcinoma after adenocarcinoma. The high background risk of non‐familial SPLC is related to the dominant role of underlying IPLC and its treatment [17, 25, 30]. Also quitting smoking upon cancer diagnosis reduces the risk for SPLC (https://www.iarc.who.int/wp‐content/uploads/2021/07/pr300_E.pdf). One can speculate that the RR of family history will increase when survival in IPLC keeps on increasing.

The role of adenocarcinoma in SPLC is dominant also because of its prevalence as the most common type of SPLC, accounting for 61% of all SPLC and for 77% of SPLC when IPLC was adenocarcinoma. The distribution of other histological types of SPLC was 19% for SCC, 9% for small cell and 11% for large cell, which deviated only little from the distribution reported in previous large studies [17, 31].

CumP of SPLC was approximately equally high for non‐familial IPLC adenocarcinoma and SCC but lower for IPLC of small cell histology (Figure 1); the data are consistent with the SEER results [31]. The low CumP for small cell carcinoma reflects poor survival for this histology. For familial SPLC, CumP increased somewhat more for SPLC after primary adenocarcinoma than after SCC. CumP of SPLC was higher for familial than for non‐familial SPLC both for men and women (Figure 2). It reached the highest level of 0.039 in 20 years of follow‐up after IPLC in female familial adenocarcinoma; the related CumP for SCC was 0.032.

Among the two main limitations of the current study, the first is case numbers, which did not allow further stratification by all important predictors, such as combinations of histology, sex and type of affected proband. The second is the lack of smoking information, which is nowhere available at the national level. Lack of this information excluded the possibility of assessing the extent of familial risk represented by shared smoking habits or quantifying its likely negative bias on SPLC due to decreased survival in smokers. Nevertheless, as reported in Methods, only 11% of currently diagnosed Swedish men and 17% of women are never smokers (earlier nonsmokers were even fewer). Thus, the lack of smoking information will be a serious future problem.

In conclusion, the 4‐fold risk of non‐familial risk of SPLC compared to IPLC and the additional 1.44‐fold risks after familial adenocarcinoma provide clinical motivations for follow‐up of patients for SPLC. Little previous data are available on the contribution of familial risk to SPLC, and the present sex‐and histology‐specific data are novel. The results support the inclusion of family history data in risk stratification models to identify patients eligible for LC screening programs (ERS/ESR factsheet).

## Author Contributions

**Frantisek Zitricky:** methodology; writing – review and editing; formal analysis. **Kristina Sundquist:** data curation; investigation; writing – review and editing; validation; resources. **Jan Sundquist:** data curation; investigation; writing – review and editing; resources. **Asta Försti:** data curation; validation; writing – original draft; writing – review and editing; visualization. **Akseli Hemminki:** investigation; writing – review and editing. **Kari Hemminki:** conceptualization; methodology; investigation; writing – original draft; writing – review and editing; project administration.

## Funding

The SALVAGE project, reg.no: CZ.02.01.01/00/22_008/0004644‐ co‐financed by the European Union and the state budget of the Czech Republic, Jane and Aatos Erkko Foundation, Sigrid Juselius Foundation, Finnish Cancer Organizations and Helsinki University Central Hospital. [Correction added on December 26, 2025 after first online publication. The funding information section has been updated in this version.]

## Ethics Statement

The study was approved by the Swedish Ethical Review Authority (Reference 2021/05188). There was no need to include informed consent in the use of nationwide register data. The study was conducted in accordance with the Declaration of Helsinki.

## Conflicts of Interest

A.H. is a shareholder in Circio Holdings ASA. A.H. is an employee and shareholder in TILT Biotherapeutics Ltd. Other authors declared no conflicts of interest.

## Data Availability

Anyone wishing to use these data should contact the National Board of Health and Welfare, Stockholm, and Statistics Sweden.
