# Correction to “Second Primary Lung Cancer Associated With Family History of Lung Cancer”

**DOI:** 10.1002/cam4.71522

**Published:** 2026-01-04

F. Zitricky, K. Sundquist, J. Sundquist, A. Försti, A. Hemminki, and K. Hemminki, “Second Primary Lung Cancer Associated With Family History of Lung Cancer,” Cancer Medicine 14, no. 23 (2025): e71431, https://doi.org/10.1002/cam4.71431.

The “Funding” section contained an incomplete funding statement:

The SALVAGE project, reg.no: CZ.02.01.01/00/22_008/0004644, Jane and Aatos Erkko Foundation, Sigrid Juselius Foundation, Finnish Cancer Organizations, and Helsinki University Central Hospital.

The text should read:

The SALVAGE project, reg. no: CZ.02.01.01/00/22_008/0004644 co‐financed by the European Union and the state budget of the Czech Republic, Jane and Aatos Erkko Foundation, Sigrid Juselius Foundation, Finnish Cancer Organizations, and Helsinki University Central Hospital.

We apologize for the error.

